# Effect of pelvic floor muscle training with or without dynamic neuromuscular stabilization–based breathing on menstrual pain and symptoms of primary dysmenorrhea: a randomized controlled trial

**DOI:** 10.1186/s12905-026-04578-w

**Published:** 2026-06-11

**Authors:** Seda Yakıt Yeşilyurt, Tansu Birinci Olgun, Yağmur Dağ, Elif Nur Dönmez, İrem Aytekin, Melis Eda Erkanlı, Adnan Budak

**Affiliations:** 1 Department of Physiotherapy and Rehabilitation, Faculty of Health Sciences, Izmir University of Economics, Izmir, Türkiye; 2 Department of Physiotherapy and Rehabilitation, Faculty of Health Sciences, Istanbul Medeniyet University, Istanbul, Türkiye; 3 Department of Obstetrics and Gynecology, University of Health Sciences, Izmir Tepecik Education and Research Hospital, Izmir, Türkiye

**Keywords:** Breathing exercises, Dysmenorrhea, Exercise therapy, Quality of life, Pain, Pelvic floor

## Abstract

**Background:**

Breathing exercises may modulate dysmenorrhea by enhancing relaxation and eliciting analgesic effects. This study investigated the effects of Pelvic Floor Muscle Training (PFMT) with or without Dynamic Neuromuscular Stabilization (DNS)-based breathing exercises on menstrual symptoms, pain, and quality of life in primary dysmenorrhea (PD).

**Methods:**

Sixty-six women with PD were randomly assigned to one of three groups: PFMT combined with DNS-based breathing exercises (PFMT + DNS Group), PFMT alone (PFMT Group), or no intervention (Control Group) (once a week for 4 weeks). Outcomes were assessed using the Menstrual Symptom Questionnaire (MSQ), the McGill Pain Questionnaire (MPQ), and the Short Form-36 (SF-36) at baseline, post-treatment, and the 3-month follow-up.

**Results:**

Both intervention groups showed significant improvements in MSQ subscales and MPQ scores from baseline to post-treatment (*P* < 0.05). Significant group × time interactions were observed for somatic complaints (*P* = 0.020, η² = 0.088), pain symptoms (*P* = 0.001, η² = 0.173), coping methods (*P* = 0.001, η² = 0.195), and total MSQ score (*P* = 0.001, η² = 0.198), and MPQ (*P* = 0.001, η² = 0.270). These improvements were maintained at the 3-month follow-up. Between-group comparisons showed significantly greater reductions in MPQ scores in both intervention groups than in control group, with no significant difference between PFMT and PFMT + DNS. No significant between-group differences were found for SF-36 subscales.

**Conclusions:**

A four-week PFMT significantly reduced menstrual symptoms and pain, with effects sustained at 3 months. However, adding DNS-based breathing exercises did not provide any additional benefits.

**Trial registration:**

ClinicalTrials.gov, Trial registration: NCT06615258, Registration date: 26 September 2024.

**Supplementary Information:**

The online version contains supplementary material available at 10.1186/s12905-026-04578-w.

## Introduction

Dysmenorrhea is a chronic menstrual-related pain typically derived from uterine cramps typically begins before menstruation and subsides after a few days [1]. It is classified as either primary or secondary dysmenorrhea [2]. Secondary dysmenorrhea is associated with identifiable gynecological conditions, most commonly, endometriosis [2, 3]. In contrast, primary dysmenorrhea (PD) is defined as painful, spasmodic lower abdominal cramps during menstruation in the absence of any identifiable organic disease [2, 4]. It is characterized by crampy, suprapubic pain that begins around menstruation, peaks with maximum blood flow, and lasts 2 to 3 days; common symptoms include nausea, vomiting, diarrhea, headache, dizziness, and fatigue [5].

Evidence-based guidelines recommend a personalized, stepwise approach to managing PD [6]. Pharmacological agents, particularly nonsteroidal anti-inflammatory drugs and hormonal therapies, are commonly used as first-line treatments; however, their use may be associated with potential side effects, including gastrointestinal discomfort and hormonal disturbances [5]. Therefore, various non-pharmacological interventions with low or no side effects have been investigated as adjunct or alternative strategies [1, 6]. Transcutaneous electrical nerve stimulation, breathing exercises, dietary modifications, therapeutic massage, local heat application, myofascial release, acupuncture, and structured exercise programs are commonly used as adjunct therapies [1, 6]. Among these approaches, pelvic floor muscle training (PFMT) has demonstrated effectiveness in reducing menstrual pain in women with PD [7, 8]. Furthermore, pain can be modulated by the relaxation-enhancing effects of breathing exercises, which are facilitated by the functional interaction between the diaphragm and the pelvic floor [9].

Dynamic Neuromuscular Stabilization (DNS) is a rehabilitative approach that integrates diaphragmatic breathing with neuromuscular control [10, 11]. DNS-based breathing exercises promote concentric diaphragm contractions, resulting in the three-dimensional expansion of the abdominal wall, and require coordinated eccentric activation of the thoracic and abdominal musculature [10]. Notably, DNS has been applied effectively to gynecological and pelvic floor dysfunctions, including stress urinary incontinence [10] and dysfunctional voiding [12], and has shown benefits in reducing pain and disability across various musculoskeletal conditions [13, 14]. However, the effects of DNS-based breathing exercises on PD are still unknown. The aim of this study, therefore, is to investigate the impacts of DNS-based breathing exercises combined with PFMT on menstrual-related symptoms, menstrual pain, and quality of life in women with PD. We hypothesized that, within a single menstrual cycle, the combined intervention would result in greater improvements in menstrual-related symptoms and in reducing menstrual pain.

## Methods

### Study design

The study was approved by the Health Sciences Research Ethics Committee of İzmir University of Economics (approval number: B.30.2.İEÜSB.0.05.05-20-297; decision no: 73; date: 12 July 2024). The trial was conducted in accordance with the ethical principles outlined in the Declaration of Helsinki. Written and verbal informed consent was obtained, indicating that their participation was voluntary. The trial was prospectively registered at ClinicalTrials.gov under the identifier NCT06615258. The study was designed, conducted, and reported in accordance with the Consolidated Standards of Reporting Trials (CONSORT) 2025 statement for randomized controlled trials.

### Setting and participants

Sixty-six women diagnosed with PD were recruited from the Department of Obstetrics and Gynecology at Izmir Tepecik Training and Research Hospital between 1 October 2024 and 1 March 2025. All participants were diagnosed with PD by an experienced gynecologist through a screening pelvic radiograph and magnetic resonance imaging (MRI) to exclude potential secondary causes such as endometriosis, fibroids, or other pelvic pathologies (AB) [5]. The eligibility criteria were as follows: (1) female participants aged 18–35 years, (2) regular menstrual cycles with a duration of 28 ± 7 days, (3) a self-reported menstrual pain intensity of > 3/10 on the Visual Analog Scale (VAS), (4) menstrual pain radiating to the back, legs, lower abdomen, or suprapubic region, (5) menstrual pain causing interference with daily activities, (6) use of medical intervention or self-medication to manage symptoms, and (7) voluntary participation with informed consent. Participants were excluded if they: (1) had a known pelvic pathology or a history of pelvic surgery, (2) had diagnosed neurological or psychological disorders, (3) had respiratory conditions that could affect chest expansion (e.g., chronic obstructive pulmonary disease, asthma, pneumonia, or bronchiectasis), (4) absence of sexual activity (no history of sexual intercourse, (5) used an intrauterine device, and (6) had a history of childbirth or miscarriage.

### Randomization

**Fig. 1:**
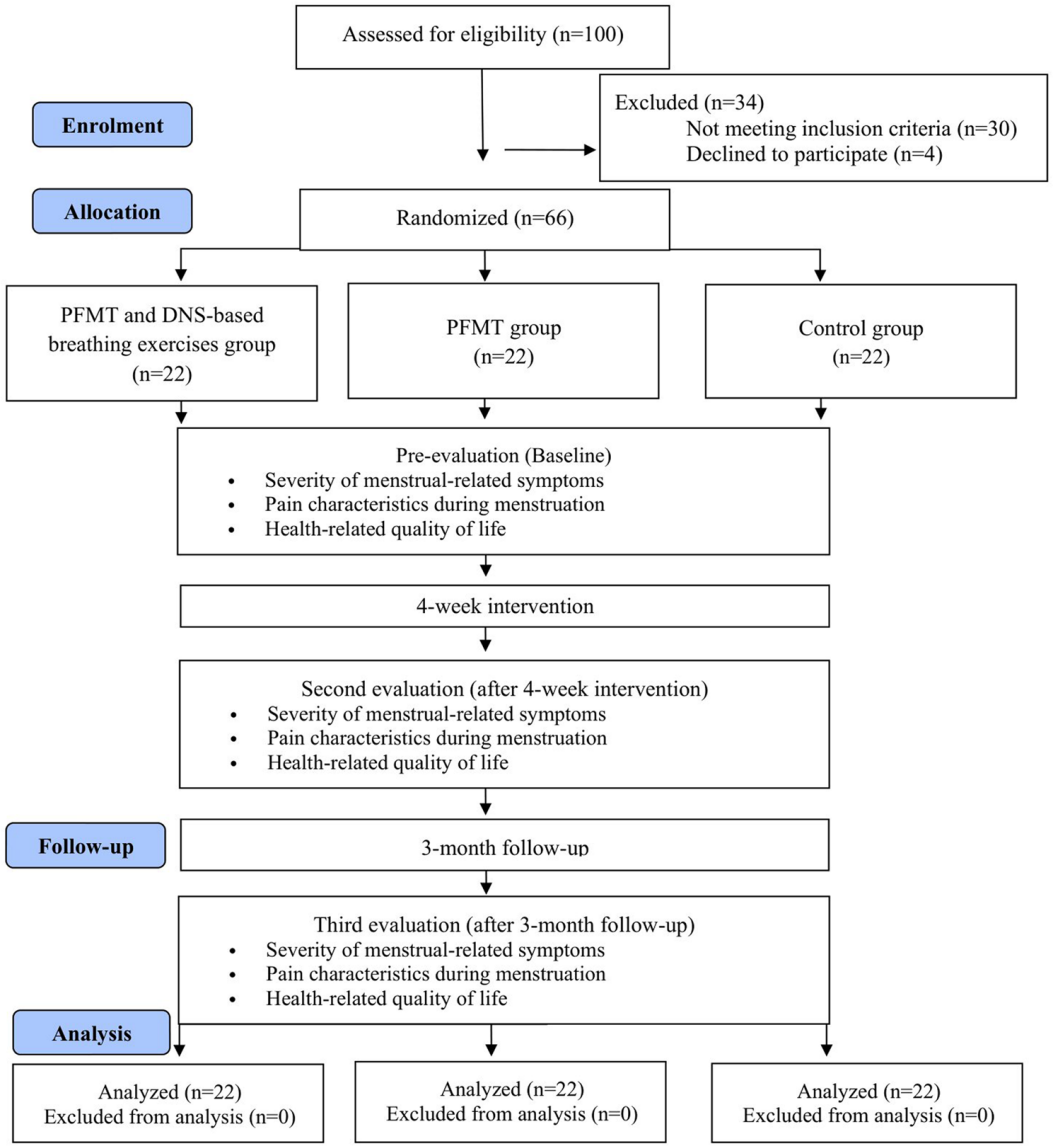
CONSORT flow diagram of the study

Among 100 women diagnosed with PD, sixty-six who met the inclusion criteria were assigned to one of three groups using a simple randomization method (1:1:1 ratio): PFMT combined with DNS-based breathing exercises (PFMT + DNS group) (*n* = 22), PFMT alone (PFMT group) (*n* = 22), or no intervention (control group) (*n* = 22) (Fig. 1). Participant allocation was carried out using a computerized randomization method (https://www.randomizer.org) by an independent researcher who was not involved in the intervention or assessment. To ensure confidentiality of allocation, group assignments were placed in sequentially numbered, sealed, opaque envelopes. The physiotherapists implemented the intervention protocols, and a separate therapist (SYY), blinded to the group allocation, conducted all outcome measurements. Data analysis was conducted independently by a third therapist (TBO).

### Outcome measures

The primary outcome measure was the Menstrual Symptom Questionnaire (MSQ), a validated self-reported instrument that assesses the frequency and severity of menstrual-related symptoms. It consists of 22 items rated on a five-point Likert scale (1 = never to 5 = always), covering three subscales: Negative Effects/Somatic Complaints (items 1–13), Menstrual Pain Symptoms (items 14–19), and Coping Methods (items 20–22). Total scores range from 22 to 110, with higher scores indicating greater symptom severity [15, 16].

Secondary outcome measures included perceived pain intensity and pain characteristics during menstruation, as well as health-related quality of life. Perceived pain intensity and characteristics during menstruation were assessed using the McGill Pain Questionnaire (MPQ), a validated multidimensional instrument that evaluates both the intensity and qualitative aspects of pain. Using this tool, participants select those descriptive words that best characterize their menstrual pain from a total of 78 words. These descriptors are categorized into sensory, affective, evaluative, and miscellaneous dimensions. Total scores range from 0 (no pain) to 78 (maximum perceived pain severity) [17, 18].

Health-related quality of life was assessed using the Short Form-36 (SF-36), a widely used, validated questionnaire that measures eight domains: physical functioning, role limitations due to physical and emotional problems, bodily pain, general health, vitality, social functioning, emotional well-being, and mental health. Scores for each domain were transformed into a 0–100 scale, with higher scores indicating better perceived health status [19, 20].

Primary and secondary outcome measures were evaluated at three time points: baseline (pre-intervention), immediately after the 4-week intervention, and at 3-month follow-up. All assessments were conducted in a clinical setting and were not taken during the menstrual period. During the follow-up period, participants in the PFMT + DNS and PFMT groups were instructed to continue their respective home-based exercise programs. To monitor adherence, each participant was provided with a personalized exercise diary containing detailed instructions on frequency, repetition counts, and postural alignment to document completion of home exercises after each session. The control group received no therapeutic input throughout the study period.

### Interventions

Participants in the PFMT + DNS group received a combined intervention comprising PFMT and DNS-based breathing exercises. In contrast, participants in the PFMT group received only PFMT, and those in the control group received no intervention. The intervention was initiated outside the menstrual phase to minimize the potential confounding effects of acute menstrual pain on exercise performance and participant adherence. All interventions were administered once weekly for a total duration of four weeks. During the follow-up period, participants in the PFMT + DNS and PFMT groups were instructed to continue their respective home-based exercise programs twice per week, following the same standardized protocol used during the supervised sessions (two sets of 10 repetitions per session). As part of the home program, participants in the PFMT + DNS group performed their breathing exercises with an elastic resistance band instead of the biofeedback belt used during supervised sessions. Participants were instructed not to use any analgesic medications throughout the study period, and adherence to this restriction was monitored through regular questioning.

The PFMT program consisted of two components: relaxation training and contraction exercises. Relaxation training was conducted in three positions: the modified butterfly pose, child pose, and deep squat position (Appendix 1) [21]. In each position, participants were instructed to actively relax the PFM for 10 s, focusing on releasing any tension without engaging surrounding muscles. This was followed by a 30-second passive rest period between repetitions, during which no specific instructions were given. Each position was repeated five times. PFMT was delivered in accordance with the exercise and intervention description components of the CERT-PFMT guideline [22]. To ensure proper muscle activation, a physiotherapist with 10 years of clinical experience guided participants through PFM contraction exercises in the side-lying position, using external palpation of the coccyx. Participants were instructed to perform isolated PFM contractions without engaging the gluteal, abdominal, or hip abductor muscles. Participants were considered to have achieved correct activation when a visible and palpable inward lift of the pelvic floor was observed without compensatory muscle activity. The physiotherapist facilitated the proper technique using metaphors such as “stopping the flow of urine” or “elevating an internal lift”. Once participants were able to perform correct contractions, exercises progressed to hook-lying, seated, and standing positions in accordance with standardized training principles. The protocol targeted both slow- and fast-twitch muscle fibers. For the former, participants were asked to perform near-maximal contractions held for 10 s, followed by 10 s of relaxation. After each sustained contraction, they performed three quick contractions. This cycle consisted of one set, and each session included three sets of 10 repetitions, with a 2-minute rest between sets [7, 23].

The DNS-based breathing training was conducted using tactile biofeedback. The targeted tactile cues were delivered using Core 360™ belts, which contained two firm foam inserts. One insert was placed anteriorly over the inguinal region, and the other was positioned posteriorly at the Grynfeltt-Lesshaft triangle [24]. The breathing exercises were performed in three developmental positions: seated, quadruped, and the “happy baby” position [25] (Appendix 2). If intra-abdominal pressure decreased or optimal alignment was disrupted, the exercise session was temporarily paused, and posture was corrected by manually reinforcing the foam pads to restore proper pressure and positioning [25]. Participants practiced each posture for 5–6 min, for a total of 20 min of DNS-based breathing training per session.

### Sample size calculation

This study was a prospective, single-blind, randomized controlled trial. The sample size calculation was performed using G^*^Power version 3.1.9.2. An F-test for repeated measures ANOVA (within–between interaction) was conducted with the following parameters: three groups, three time points, a significance level (α) of 0.05, a power of 95% (1–β = 0.95), and a medium effect size (f = 0.25) [26]. The analysis indicated a minimum of 18 participants per group was required. To account for a potential dropout rate of approximately 20%, 66 participants were recruited to achieve a sufficient sample size.

### Statistical analysis

All statistical analyses were performed using the Statistical Package for the Social Sciences (SPSS) for Windows, version 21.0 (SPSS Inc., Chicago, IL, USA). All analyses were performed according to the intention-to-treat (ITT) principle, with participants analyzed in the groups to which they were originally randomized. As there were no missing data or losses to follow-up, no imputation methods were required. The normality of data distribution was assessed using the Kolmogorov–Smirnov test. Baseline demographic and clinical variables were compared among the three groups using one-way analysis of variance (ANOVA) for continuous variables and the Chi-square test for categorical variables. Within-group comparisons (from baseline to post-treatment and follow-up) were evaluated using paired sample t-tests. To control for multiple comparisons, a Bonferroni correction was applied, and the significance level was set at *p* < 0.025. The two-way repeated-measures ANOVA was conducted with time (baseline, post-treatment, and 3-month follow-up) as the within-subjects factor and group (PFMT + DNS group, PFMT group, and control group) as the between-subjects factor to examine changes over time and differences between groups. When significant main or interaction effects were detected, Bonferroni-adjusted post hoc pairwise comparisons were performed. A *P* value of < 0.05 was considered statistically significant. Effect sizes were calculated using partial eta squared (η²) for ANOVA tests, interpreted as small (0.01), medium (0.06), and large (0.14) effects according to Cohen’s criteria [26].

## Results

A total of 100 women with PD were screened for eligibility. Thirty-four did not meet the inclusion criteria; thus, 66 women with PD completed the study and were included in the analysis. All randomized participants completed the study and were included in the final analysis. No participants were lost to follow-up, and no data were missing (Fig. 1). There were no statistically significant differences between the groups in terms of demographic variables, clinical parameters, or outcome measures at baseline (*P* > 0.05 for all comparisons) (Table 1). During the intervention period, participants in the PFMT + DNS group reported completing a mean of 4.55 home exercise sessions, whereas participants in the PFMT group completed 5.73 sessions (*P* = 0.542). No intervention-related adverse events or unintended effects were reported in any study group during the intervention or follow-up period.

**Table 1:** Baseline and clinical characteristics of patients wıth primary dysmenorrhea among study groups

	Group 1 (*N* = 22)	Group 2 (*N* = 22)	Group 3 (*N* = 22)	*P* ^*^
Age (yr), mean (SD)	21.32 (2.12)	22.41 (3.45)	21.77 (1.23)	0.332
Body mass index (kg/m^2^), mean (SD)	21.40 (3.65)	21.48 (5.17)	21.80 (3.88)	0.945
Active sexual life, n (%)				
Presence	2 (9.1)	5 (22.7)	4 (18.2)	0.467^β^
Absence	20 (90.9)	17 (77.3)	18 (81.8)	
Menarche age (yr), mean (SD)	13.36 (1.00)	13.09 (1.71)	13.05 (1.67)	0.751
Sanitary pad usage, mean (SD)	11.36 (3.27)	11.64 (2.23)	11.23 (2.67)	0.884
Period pain intensity (points), mean (SD)	7.73 (1.60)	8.57 (1.43)	7.95 (1.67)	0.198
Bleeding duration (days), mean (SD)	5.27 (1.20)	5.50 (0.96)	5.68 (1.24)	0.494
MSQ (score), mean (SD)				
Somatic complaints	47.55 (10.22)	47.59 (6.93)	47.91 (9.38)	0.982
Pain symptoms	23.14 (4.38)	23.41 (2.92)	22.27 (2.88)	0.526
Coping methods	11.91 (1.92)	11.32 (3.63)	10.41 (2.32)	0.193
Total	81.50 (13.73)	81.41 (9.59)	78.95 (13.13)	0.738
MPQ (score), mean (SD)	34.68 (15.24)	37.91 (19.02)	40.2 (13.09)	0.511
S-36 (score), mean (SD)				
Physical functioning	87.27 (12.79)	82.95 (26.44)	77.50 (21.31)	0.301
Role physical	72.72 (31.72)	68.18 (38.71)	66.81 (43.08)	0.364
Bodily pain	58.52 (18.86)	56.70 (29.51)	54.20 (16.19)	0.817
General health	58.40 (16.85)	65.90 (15.78)	58.86 (16.89)	0.243
Vitality	52.50 (15.71)	52.50 (19.86)	47.09 (17.44)	0.512
Social functioning	65.34 (18.47)	67.04 (23.32)	63.63 (23.75)	0.875
Role emotional	39.39 (8.39)	40.35 (8.60)	45.29 (9.65)	0.313
Emotional well-being	61.27 (11.67)	59.27 (18.41)	53.45 (18.78)	0.276

Group 1: Pelvic floor muscle training combined with dynamic neuromuscular stabilization-based breathing exercises; Group 2: Pelvic floor muscle training; Group 3: Control group

Data presented as number (percentage) and mean (standard deviation) as appropriate

*Abbreviations*: *%* percentage, *BMI* Body mass index, *MPQ* McGill Pain Questionnaire, *MSQ* Menstrual Symptom Questionnaire, *N* number of participants, *SD* standard deviation, *SF-36* Short Form-36, *BMI* body mass index, is weight in kilograms divided by the square of height in meters

^*^One-way analysis of variance (one-way ANOVA); significance level set at  < 0.05

^β^Chi-squared test; significance level set at  < 0.05

### Changes in MSQ subscale scores

**Fig. 2:**
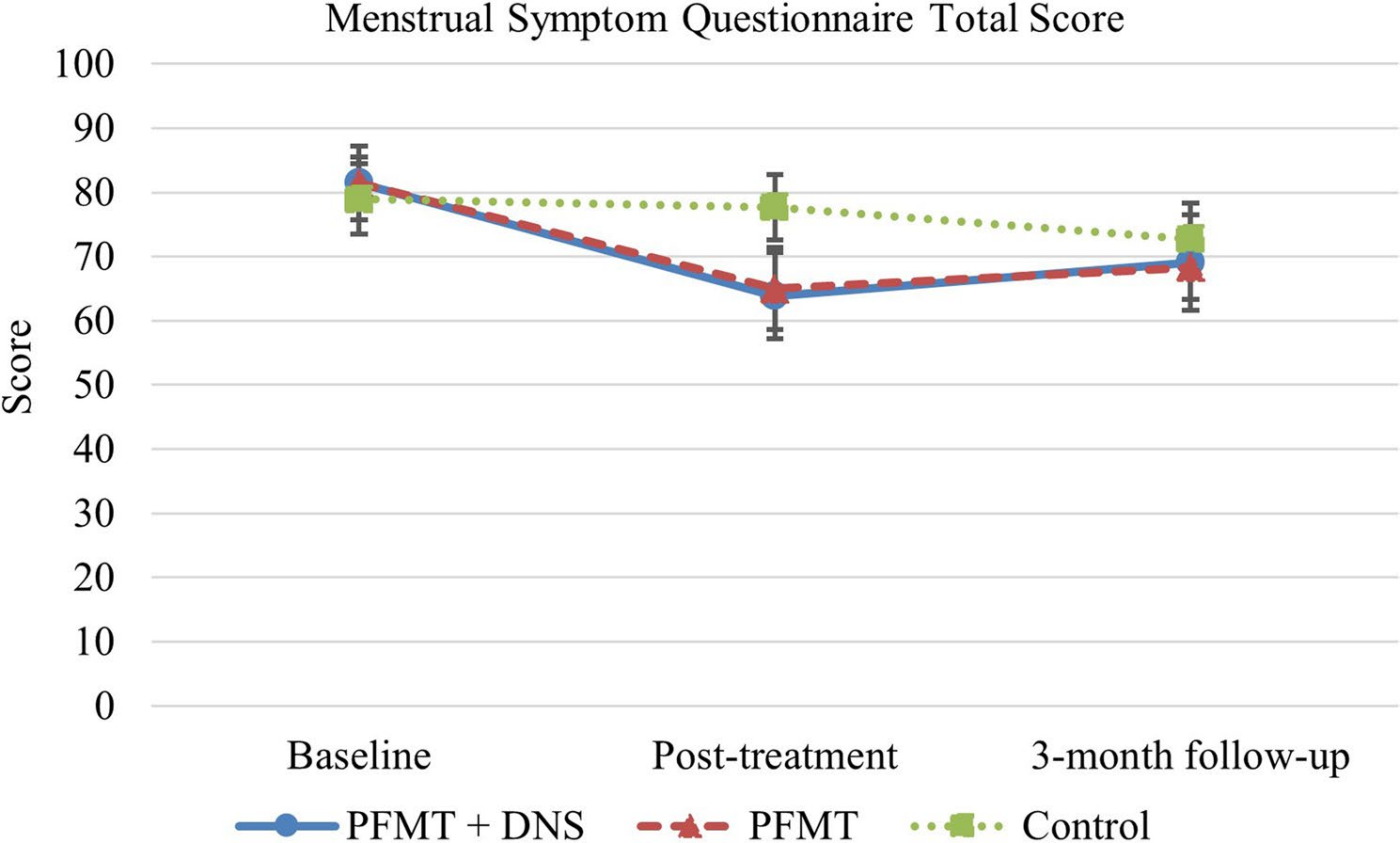
Changes in MSQ total score over time across groups. Data are presented as mean values with 95% confidence intervals. Two-way repeated-measures ANOVA revealed a significant group × time interaction effect (F = 7.767, *P* = 0.001, η² = 0.198)

Within-group analyses demonstrated statistically significant improvements in all MSQ subscales (somatic complaints, pain symptoms, and coping methods) in both the PFMT + DNS and PFMT groups from baseline to post-treatment (*P* < 0.05), and these improvements were maintained at the 3-month follow-up. A significant group × time interaction was observed for somatic complaints (F = 3.033, *P* = 0.020, η² = 0.088), pain symptoms (F = 6.606, *P* = 0.001, η² = 0.173), coping methods (F = 7.630, *P* = 0.001, η² = 0.195), and total MSQ score (F = 7.767, *P* = 0.001, η² = 0.198), indicating that changes over time differed significantly between groups (Table 2, Fig. 2). Although significant group × time interaction effects were observed, Bonferroni-adjusted pairwise comparisons did not demonstrate statistically significant differences between groups in any MSQ subscale (Table 2).

**Table 2:** Within- and between-group changes in menstrual symptom severity and pain outcomes over time in patients with primary dysmenorrhea

Variables	Group	Baseline	Post-treatment	Change from baseline to the post-treatment^a^	3-month follow-up	Change from baseline to follow-up^a^	*P* ^b^	Between-group mean difference (95% CI)		*P* ^c^
MSQ Somatic complaints	**Group 1**	47.55 (10.22)	38.00 (9.96)	**-9.54 [-11.72**,** -11.59]**^**a**^	40.45 (10.30)	**-7.09 [-9.99**,** -4.27]**^**a**^	**0.020**	G1-G2	0.16 (-5.78, 6.12)	1.00
	**Group 2**	47.59 (6.93)	38.55 (9.33)	**-9.04 [-13.58**,** -5.09]**^**a**^	39.36 (8.01)	**-8.22 [-11.04**,** -5.77]**^**a**^		G1-G3	-3.36 (-9.31, 2.59)	0.50
	**Group 3**	47.91 (9.38)	45.14 (8.12)	-2.77 [-5.77, 0.54]	43.05 (8.93)	-4.86 [-7.45, -2.45]		G2-G3	-3.53 (-9.48, 2.42)	0.44
MSQ Pain symptoms	**Group 1**	23.14 (4.38)	17.55 (4.63)	**-5.59 [-7.18**,** -4.00]**^**a**^	19.64 (5.84)	**-3.50 [-5.50**,** -1.40]**^**a**^	**0.001**	G1-G2	-0.45 (-3.01, 2.10)	1.00
	**Group 2**	23.41 (2.92)	18.32 (4.36)	**-5.09 [-6.81**,** -3.50]**^**a**^	19.95 (3.49)	**-3.45 [-4.45**,** -2.54]**^**a**^		G1-G3	-1.42 (-3.98, 1.13)	0.52
	**Group 3**	22.27 (2.88)	21.95 (3.82)	-0.31 [-1.77, 1.04]	20.36 (3.68)	-1.90 [-3.36, -0.40]		G2-G3	-0.97 (-3.52, 1.58)	1.00
MSQ Coping methods	**Group 1**	11.91 (1.92)	8.32 (3.16)	**-3.59 [-4.90**,** -2.31]**^**a**^	9.09 (3.43)	**-2.81 [-4.13**,** -1.50]**^**a**^	**0.001**	G1-G2	0.37 (-1.55, 2.31)	1.00
	**Group 2**	11.32 (3.63)	7.91 (3.35)	**-3.40 [-4.59**,** -2.22]**^**a**^	8.95 (3.10)	**-2.36 [-3.22**,** -1.36]**^**a**^		G1-G3	-0.42 (-2.36, 1.51)	1.00
	**Group 3**	10.41 (2.32)	10.41 (2.55)	0.00 [-0.50, 0.45]	9.77 (2.81)	-0.63 [-1.40, 0.09]		G2-G3	-0.80 (-2.74, 1.13)	0.93
MSQ Total	**Group 1**	81.50 (13.73)	63.86 (16.01)	**-17.63 [-21.40**,** -13.95]**^**a**^	69.05 (17.78)	**-12.45 [-17.63**,** -7.59]**^**a**^	**0.001**	G1-G2	-0.91 (-9.24, 9.06)	1.00
	**Group 2**	81.41 (9.59)	65.00 (15.38)	**-16.40 [-22.04**,** -10.86]**^**a**^	68.27 (11.88)	**-13.13 [-16.72**,** -9.22]**^**a**^		G1-G3	-4.98 (-14.14, 4.17)	0.55
	**Group 3**	78.95 (13.13)	77.68 (12.23)	-1.27 [-3.95, 1.63]	72.73 (13.33)	-6.22 [2.13, 11.00]		G2-G3	-4.89 (-14.05, 4.26)	0.58
Pain quality and intensity										
MPQ	**Group 1**	34.68 (15.24)	14.82 (12.41)	**-19.86 [-26.63**,** -13.04]**^**a**^	19.64 (13.20)	**-15.04 [-22.63**,** -8.04]**^**a**^	**0.001**	G1-G2	-5.24 (-14.44, 3.96)	0.49
	**Group 2**	37.91 (19.02)	20.45 (15.51)	**-17.45 [-23.59**,** -11.59]**^**a**^	26.50 (17.47)	**-11.40 [-19.90**,** -3.09]**^**a**^		G1-G3	-21.62 (-30.82, -12.41)	**0.001**
	**Group 3**	40.27 (13.09)	48.32 (14.43)	**8.04 [2.09**,** 13.90]**^**a**^	45.41 (18.89)	5.13 [-1.54, 11.09]		G2-G3	-16.00 (-25.58, -7.17)	**0.001**

Group 1 (G1): Pelvic floor muscle training combined with dynamic neuromuscular stabilization-based breathing exercises; Group 2 (G2): Pelvic floor muscle training; Group 3 (G3): control group

Data are presented as mean ± standard deviation and mean differences (95% confidence interval [lower, upper])

*Abbreviations*: *%* percentage, *CI* confidence interval, *MPQ* McGill Pain Questionnaire, *MSQ* Menstrual Symptom Questionnaire

ᵃ Within-group comparisons (baseline vs. post-treatment and baseline vs. follow-up) were performed using paired-samples t-tests. To control for multiple comparisons, the Bonferroni-adjusted significance level was set at *p* < 0.025

ᵇ *p*-values represent the significance of the group × time interaction effect obtained from two-way repeated-measures ANOVA. Statistical significance was set at *p* < 0.05, and statistically significant results are shown in bold

ᶜ *p*-values represent Bonferroni-adjusted pairwise comparisons between groups; statistical significance was set at *P* < 0.05, and statistically significant differences are shown in bold

### Changes in MPQ score

**Fig. 3:**
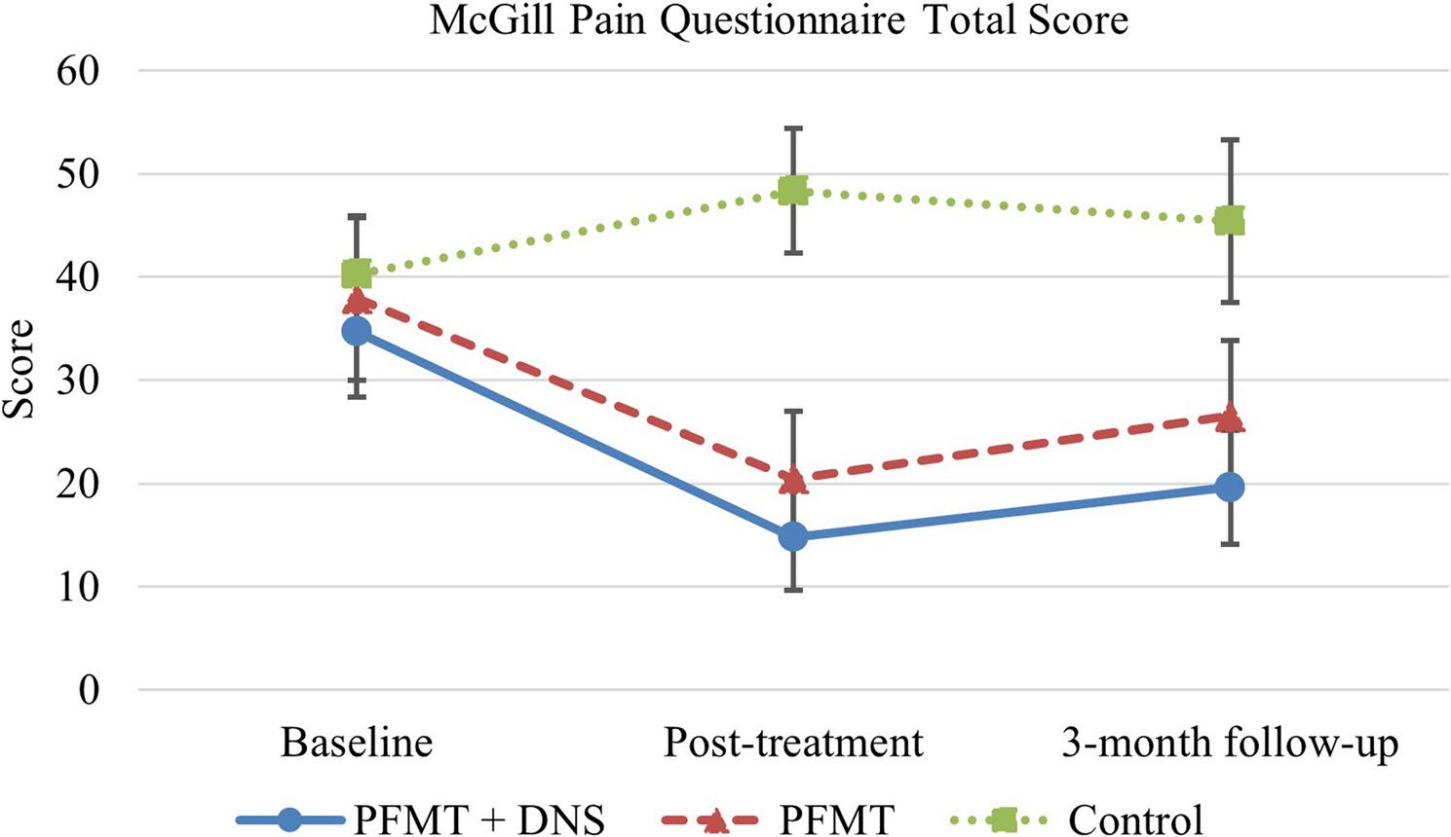
Changes in MPQ total score over time across groups. Data are presented as mean values with 95% confidence intervals. Two-way repeated-measures ANOVA revealed a significant group × time interaction effect (F = 11.661, *P* = 0.001, η² = 0.270)

Within-group analyses demonstrated statistically significant reductions in MPQ score in both the PFMT + DNS and PFMT groups from baseline to post-treatment (*P* < 0.05), and these improvements were maintained at the 3-month follow-up. In contrast, the control group showed an increase in MPQ scores over time. A significant group × time interaction effect for MPQ scores (F = 11.661, *P* = 0.001, η² = 0.270), indicating that changes in pain severity over time differed significantly between groups (Table 2, Fig. 3). Bonferroni-adjusted pairwise comparisons showed that both intervention groups demonstrated significantly greater improvements than the control group at follow-up. In contrast, no significant difference was observed between the PFMT + DNS and PFMT groups (Table 2).

### Changes in SF-36 subscale scores

Within-group analyses showed no significant improvements in several SF-36 subscales across both intervention groups and the control group. Consistently, two-way repeated-measures ANOVA revealed no significant group × time interaction effects for any SF-36 subscale (*P* > 0.05) (Table 3).

**Table 3:** Within- and between-group changes in health-related quality of life over time in patients with primary dysmenorrhea

Variables	Group	Baseline	Post-treatment	Change from baseline to the post-treatment^a^	3-month follow-up	Change from baseline to follow-up^a^	*P* ^b^
Health-related quality of life							
SF-36 Physical functioning	**Group 1**	87.27 (12.79)	88.86 (11.01)	1.59 [-2.95, 5.68]	89.63 (12.81)	2.36 [-3.77, 8.40]	0.234
	**Group 2**	82.95 (26.44)	88.48 (16.02)	5.53 [-4.68, 16.74]	92.72 (15.48)	9.77 [-0.45, 20.44]	
	**Group 3**	77.50 (21.31)	71.81 (26.88)	-5.68 [-15.68, 0.90]	81.81 (19.67)	4.31 [-1.81, 9.54]	
SF-36 Role physical	**Group 1**	72.72 (31.72)	77.72 (30.42)	5.00 [-7.95, 20.45]	72.04 (37.02)	-0.68 [-18.18, 17.50]	0.160
	**Group 2**	68.18 (38.71)	72.04 (19.49)	3.86 [5.68–17.20]	75.22 (30.53)	7.04 [3.40-28.63]	
	**Group 3**	66.81 (43.08)	63.42 (42.46)	-3.40 [-21.59, 13.63]	61.13 (42.78)	5.68 [-15.90, 27.27]	
SF-36 Bodily pain	**Group 1**	58.52 (18.86)	68.50 (18.77)	9.97 [-0.15, 19.77]	63.40 (19.26)	4.88 [-5.00, 14.53]	0.194
	**Group 2**	56.70 (29.51)	68.98 (16.46)	12.28 [2.06, 26.27]	61.81 (14.92)	5.11 [-10.89, 19.65]	
	**Group 3**	54.20 (16.19)	58.39 (21.62)	4.81 [-10.56, 7.61]	58.18 (22.61)	3.97 [-6.02, 14.76]	
SF-36 General health	**Group 1**	58.40 (16.85)	58.32 (13.14)	-0.08 [-6.36, 5.90]	60.68 (14.74)	2.27 [-3.17, 7.27]	0.283
	**Group 2**	65.90 (15.78)	66.13 (13.70)	0.22 [-4.54, 5.74]	67.95 (12.40)	2.04 [-3.40, 7.95]	
	**Group 3**	58.86 (16.89)	57.95 (15.24)	-0.90 [-5.00, 2.95]	54.31 (13.02)	-4.54 [-9.77, -0.22]	
SF-36 Vitality	**Group 1**	52.50 (15.71)	55.38 (16.76)	2.88 [-4.83, 10.22]	52.30 (16.39)	-0.19 [-7.95, 7.19]	0.699
	**Group 2**	52.50 (19.86)	57.96 (16.01)	5.46 [2.72–12.97]	56.36 (14.65)	3.86 [5.67–13.18]	
	**Group 3**	47.09 (17.44)	45.45 (15.87)	-1.63 [-9.22, 5.17]	43.40 (15.76)	-3.68 [-12.54, 4.77]	
SF-36 Social functioning	**Group 1**	65.34 (18.47)	75.00 (16.36)	9.65 [0.58, 18.75]	69.54 (20.03)	4.20 [-4.88, 13.63]	0.108
	**Group 2**	67.04 (23.32)	77.61 (17.80)	10.56 [0.68–20.22]	75.90 (14.44)	8.86 [4.54–21.92]	
	**Group 3**	63.63 (23.75)	61.36 (22.79)	-2.27 [-10.78, 6.25]	55.68 (19.94)	-7.95 [-15.32, -1.13]	
SF-36 Role emotional	**Group 1**	39.39 (8.39)	57.80 (45.11)	18.40 [0.05, 36.59]	56.21 (46.48)	16.81 [-4.55, 36.81]	0.236
	**Group 2**	40.35 (8.60)	62.31 (34.59)	21.96 [-1.35, 31.81]	57.16 (29.61)	16.81 [-4.55, 26.81]	
	**Group 3**	45.29 (9.65)	54.55 (44.29)	9.08 [-10.62, 31.80]	46.96 (45.61)	1.49 [-22.69, 25.72]	
SF-36 Emotional well-being	**Group 1**	61.27 (11.67)	59.81 (13.99)	-1.45 [-7.81, 5.09]	58.22 (15.67)	-3.04 [-10.36, 5.00]	0.367
	**Group 2**	59.27 (18.41)	63.77 (13.12)	4.50 [3.08, 12.04]	58.36 (13.92)	-0.90 [-8.17, 10.67]	
	**Group 3**	53.45 (18.78)	53.27 (19.01)	-0.18 [-8.00, 6.18]	52.00 (13.96)	-1.45 [-9.26, 6.18]	

Group 1 (G1): Pelvic floor muscle training combined with dynamic neuromuscular stabilization-based breathing exercises; Group 2 (G2): Pelvic floor muscle training; Group 3 (G3): control group

Data are presented as mean ± standard deviation and mean differences (95% confidence interval [lower, upper])

*Abbreviations*: *%* percentage, *CI* Confidence interval, *SF-36* Short Form-36

ᵃ Within-group comparisons (baseline vs. post-treatment and baseline vs. follow-up) were performed using paired-samples t-tests. To control for multiple comparisons, the Bonferroni-adjusted significance level was set at *p* < 0.025

ᵇ *p*-values represent the significance of the group × time interaction effect obtained from two-way repeated-measures ANOVA. Statistical significance was set at *p* < 0.05

## Discussion

The findings suggest that PFMT, applied alone or combined with DNS-based breathing exercises, was associated with significant reductions in menstrual-related symptoms and pain in women with PD, and these effects were sustained at 3-month follow-up. These improvements were supported by moderate to large effect sizes for MSQ subscales, including somatic complaints (η² = 0.088), pain symptoms (η² = 0.173), coping methods (η² = 0.195), total score (η² = 0.198), and MPQ score (η² = 0.270). Both intervention groups demonstrated significantly greater reductions in MPQ scores compared with the control group, with mean differences of − 21.62 (95% CI: −30.82 to − 12.41) for PFMT + DNS and − 16.00 (95% CI: −25.58 to − 7.17) for PFMT alone. However, no significant difference was observed between the PFMT + DNS and PFMT groups (mean difference: −5.24; 95% CI: −14.44 to 3.96; *p* = 0.49), suggesting that the addition of DNS-based breathing did not confer a statistically significant advantage over PFMT alone in reducing pain. In addition, the intervention demonstrated only a limited impact on health-related quality of life.

Recommendations for managing menstrual pain in PD include various therapeutic modalities, such as transcutaneous electrical nerve stimulation, breathing exercises, dietary modifications, therapeutic massage, local heat application, myofascial release, acupuncture, and structured exercise programs [1, 6, 27]. Systematic reviews and clinical guidelines for the management of pelvic floor dysfunctions support PFMT. However, despite the current evidence, few studies have explored the isolated effects of PFMT specifically on menstrual symptoms and pain in young women with PD [7, 28]. A recent study comparing manual therapy, PFM exercises, and their combination in women with PD reported significant improvements in reducing pain and symptoms in all groups [7]. Our study similarly showed that PFMT, with or without DNS-based breathing exercises, improved menstrual symptoms and pain, which were sustained at the 3-month follow-up. In our study, both groups focused on PFM contraction and relaxation via PFMT. Exercises targeting the PFM specifically can lead to significant improvements in pain and menstrual symptoms by increasing muscle strength [27, 29, 30]. Moreover, yoga poses are a safe and effective treatment for PD [31, 32]. In our study, we therefore used modified yoga poses recommended in the literature, which are reported to facilitate pelvic floor relaxation for PFM during relaxation training [21]. PFMT was particularly effective in improving control of both PFM contraction and relaxation. Since PFM contraction involves both concentric and isometric components, training requires precise neuromuscular coordination [33]. Isometric exercises may affect dysmenorrhea by enhancing pelvic muscle function and potentially facilitating menstrual blood flow, which could potentially influence uterine activity [27]. In our study, the primary aim was to investigate immediate changes in menstrual-related symptoms over a single cycle, while providing sufficient overall training exposure through a combination of supervised and home exercises. Exercise studies on PD have shown that structured physical activity and relaxation‑based exercise programs can reduce menstrual pain within 4 weeks. Current evidence indicates that relaxation‑type exercise interventions significantly reduce menstrual pain after 4 and 8 weeks of practice [32]. Supervised sessions were reinforced with a structured home‑based exercise program to enhance overall training exposure.

The efficacy of PFMT is well established in the management of pelvic floor dysfunctions. However, there is also growing interest in incorporating complementary modalities such as diaphragmatic breathing into standard PFM exercise programs [32, 34]. This rationale is supported by biomechanical hypotheses that suggest fascial connections between the diaphragm and the pelvic floor [35]. Moreover, it has been proposed that deep-breathing exercises activate pain-inhibitory systems in PD, thereby promoting muscle relaxation and reducing pain, as reported among physiotherapy students with dysmenorrhea [36]. However, the findings are mixed: a recent meta-analysis and systematic review of multi-component exercise interventions found that relaxation exercises (including breathing, meditation, or similar techniques) were among the least effective modalities for PD [37]. Consistent with this, the present study found no additional benefits for symptoms, pain, or quality of life from adding DNS-based breathing exercises to PFMT. The differences in findings between the current and previous studies may be due to the use of expansive diaphragmatic exercises with biofeedback belts across three developmental kinesiological positions [36]. Our findings are consistent with a systematic review indicating that PFMT alone is significantly more effective than breathing exercises in improving PFM function and managing pelvic floor disorders, with no additional benefit observed when PFMT is combined with breathing exercises [34]. Neurophysiological studies suggest that DNS interventions may be associated with activation of brain regions such as the anterior cingulate cortex, a region crucial for motor learning, core stabilization, and respiratory control [38].

The addition of DNS-based breathing exercises was hypothesized to provide additional benefits; however, the observed differences between the PFMT and PFMT + DNS groups were modest for menstrual symptoms, pain, and health-related quality of life. Several factors may explain these findings. First, the intervention duration was limited to a single four-week menstrual cycle, which may have been insufficient to elicit clinically meaningful neuromuscular adaptations associated with DNS-based breathing. Such adaptations, particularly those involving motor control, core stabilization, and breathing pattern integration, typically require longer intervention periods and higher training frequency [39]. Second, both groups received PFMT, which is a well-established and effective intervention for improving pelvic floor function and related symptoms [7, 8]. This may have led to a ceiling effect, thereby attenuating the relative contribution of DNS-based breathing, since PFMT alone can produce substantial improvements. Taken together, these findings suggest that while DNS-based breathing may provide additional benefits, longer intervention durations and higher training frequencies may be required to fully realize its therapeutic potential.

The improvement in pain-related outcome measures did not translate into improvements in health-related quality of life. This outcome may reflect the multifactorial nature of pain, including psychosocial influences, habitual behavioral patterns, measurement tool sensitivity, and contextual factors such as mood, anticipatory anxiety, and social functioning [40]. Several studies have indicated that even after pain decreases, women with PD may continue to experience reduced quality of life due to ongoing behavioral limitations and emotional distress [1, 6]. Importantly, the use of a generic health-related quality of life measure may have limited the ability to detect changes specific to menstrual health. Menstruation-specific quality of life measures may be more sensitive in capturing subtle but clinically relevant changes related to menstrual symptoms and their impact on daily functioning. Furthermore, the duration of the intervention may have been insufficient to produce measurable improvements in health-related quality of life, consistent with previous reports that emphasize the need for longer-term interventions.

To our knowledge, this is among the first randomized controlled trials to investigate the effectiveness of PFMT both alone and in combination with DNS-based breathing exercises on menstrual symptoms, pain, and quality of life in women with PD. A notable strength of this study is the clearly defined, replicable intervention protocols, which enhance methodological transparency and may support future clinical application. The use of validated outcome measures and a follow-up period enabled the evaluation of both short-term and long-term effects.

### Limitations of the study

Several limitations of this study should be acknowledged. First, the use of a health-related quality of life measure may not have been sufficiently sensitive to detect changes specific to menstrual health. In addition, all primary and secondary outcomes were assessed using self-reported questionnaires, which may be subject to recall bias and subjective interpretation. Second, the four-week intervention period, corresponding to a single menstrual cycle, may have limited the ability to sustain neuromuscular adaptations and to establish longer-term changes. Exercise-based interventions targeting motor control and breathing patterns typically require longer durations and repeated practice to achieve stable and transferable adaptations. Third, although a 3-month follow-up was included, this period may still be considered relatively short to determine the long-term sustainability of treatment effects, particularly for interventions aimed at modifying neuromuscular control and habitual movement patterns. Fourth, the relatively young and homogeneous sample (18–35 years) may limit the generalizability of the findings to older women and more diverse populations. In addition, the absence of an active control group may limit the ability to distinguish specific treatment effects from non-specific factors such as participant attention, expectation, or engagement. Fifth, although outcome assessors were blinded to group allocation, participants were aware of their assigned interventions, which may have introduced expectation or performance bias. Finally, pelvic floor muscle activation was assessed using external palpation rather than objective measures such as electromyography, which may limit the precision of muscle activation assessment, although this approach was preferred to ensure participant comfort.

## Conclusion

This study demonstrates that 4-week PFMT is effective in reducing menstrual symptoms and pain in women with PD, with benefits sustained at three months. The addition of DNS-based breathing exercises did not result in additional benefits over PFMT alone, suggesting that PFMT may be a sufficient and efficient intervention in this population. Despite significant improvements in pain and symptom severity, no meaningful changes were observed in health-related quality of life, highlighting the potential limitations of using generic assessment tools. The short intervention duration may have constrained the detection of DNS-related motor learning effects and longer-term adaptations. Future studies should incorporate longer intervention durations, menstruation-specific health-related quality of life instruments, neurophysiological assessments, and active control groups to more precisely characterize treatment-specific effects and underlying mechanisms.

## Supplementary Information

Supplementary Material 1.

Supplementary Material 2.

## Data Availability

The datasets generated during and/or analyzed during the current study are available from the corresponding author on reasonable request.
