# Supplementary material for: Effect of pelvic floor muscle training with or without dynamic neuromuscular stabilization–based breathing on menstrual pain and symptoms of primary dysmenorrhea: a randomized controlled trial
Source: BMC Womens Health. 2026 Jun 11;26:402. doi: 10.1186/s12905-026-04578-w (PMC13479574; doi:10.1186/s12905-026-04578-w)
Supplement: Supplementary file 1 [file 12905_2026_4578_MOESM1_ESM.docx]

**Appendix 1** Pelvic floor muscle relaxation training protocol

| **Pelvic Floor Muscle Relaxation Training** | |
| --- | --- |
| During pelvic floor muscle relaxation training in the *modified butterfly position*, participants were given the following instructions:  Lie on your back with the soles of your feet together and your knees gently falling outward, allowing your hips to open. Place a pillow under each knee to support your legs and reduce tension in the inner thighs. Rest your arms comfortably at your sides. As you breathe in through your nose, feel your abdomen gently rise. As you inhale, imagine the area between your hips softening, the pelvic floor softening, and releasing downward. Stay relaxed and supported throughout the exercise. |  |
| During pelvic floor muscle relaxation training in the *child pose position*, participants were given the following instructions:  Begin in a crawling position on your hands and knees. Gently bring your forearms to the floor for support, allowing your hips to shift back and rest toward your heels. Keep the tops of your feet flat on the floor with the toes touching each other. Let your knees open wider than your hips, creating space for your abdomen to relax downward. As you settle into the position, breathe deeply through your nose. As you inhale, imagine the area between your hips softening, the pelvic floor softening, and releasing downward. Stay relaxed and supported throughout the exercise. |  |
| During pelvic floor muscle relaxation training in the *deep squat position*, participants were given the following instructions:  Stand with your feet slightly wider than hip-width apart and slowly lower yourself into a deep squat, keeping your heels in contact with the ground. Let your knees fall open comfortably, and bring your hands together in front of your chest or rest your elbows gently inside your knees for support. Keep your spine upright and your shoulders relaxed. As you inhale through your nose, feel your belly expand. As you inhale, imagine the area between your hips softening, the pelvic floor softening, and releasing downward. Stay relaxed and supported throughout the exercise. |  |
