# Supplementary material for: Effect of pelvic floor muscle training with or without dynamic neuromuscular stabilization–based breathing on menstrual pain and symptoms of primary dysmenorrhea: a randomized controlled trial
Source: BMC Womens Health. 2026 Jun 11;26:402. doi: 10.1186/s12905-026-04578-w (PMC13479574; doi:10.1186/s12905-026-04578-w)
Supplement: Supplementary file 2 [file 12905_2026_4578_MOESM2_ESM.docx]

**Appendix 2** Dynamic neuromuscular stabilization–based breathing exercise protocol

| **Dynamic Neuromuscular Stabilization–Based Breathing Exercise** | |
| --- | --- |
| During breathing training in the *seated position*, participants were given the following instructions:  Keep your spine upright. Avoid slouching your back or over-arching your lower back. Relax your shoulders. Ensure both feet are firmly planted on the floor for support. Gently push your abdominal wall against both foam pads and try to maintain steady pressure during both inhalation and exhalation. Now, breathe in and out through your nose. |  |
| During breathing training in the *quadriped position*, participants were given the following instructions:  Come onto your hands and knees. Keep your hands directly under your shoulders and your knees under your hips. Let your weight rest gently on the outer edges of your hands and on the front of your lower legs. Keep your back flat and your neck relaxed. Now, breathe in through your nose and let your belly expand gently into the belt. Feel the pressure at the front and back of your waist. Maintain that pressure as you breathe out slowly through your nose. |  |
| During breathing training in the *happy baby*, participants were given the following instructions:  Lie on your back and bring your knees toward your chest. Use your hands to hold your ankles or lower legs gently. Keep your lower back softly touching the ground and don’t let it arch or lift off the ground. Now, gently push your belly outward against the front and back pads of the belt. Try to feel the same pressure in both directions. Maintain that pressure as you breathe in and out through your nose slowly and steadily. |  |
